# Genomic Insights into *Vibrio parahaemolyticus* from Southern Korea: Pathogenicity, Antimicrobial Resistance, and Phylogenetic Distinctions

**DOI:** 10.3390/microorganisms12122497

**Published:** 2024-12-04

**Authors:** Hyunwoo Zin, Jaewon Lim, Younhee Shin, Byeori Kim, Minchul Yoon, Kwangsoo Ha, Sunghyun Cho

**Affiliations:** 1 Food Safety and Processing Research Division, National Institute of Fisheries Science, 216 Gijang-haeanro, Gijang-eup, Busan 46083, Republic of Korea; traveler10233@gmail.com (B.K.); yoonmc@korea.kr (M.Y.); ksha@korea.kr (K.H.); 2 Research and Development Center, Insilicogen Inc., 13, Yongin-si 16954, Republic of Korea; jwlim@insilicogen.com (J.L.); yhshin@insilicogen.com (Y.S.)

**Keywords:** *Vibrio parahaemolyticus*, antimicrobial resistance, Korea, multilocus sequence typing, phylogenetic analysis, whole genome sequencing

## Abstract

*Vibrio parahaemolyticus*, a significant cause of gastroenteritis and a growing public health concern, has become increasingly prevalent owing to the rise in ocean temperatures driven by climate change. This study aimed to characterize the genetic diversity, pathogenic potential, and antimicrobial resistance (AMR) profiles of *V. parahaemolyticus* strains isolated from the southern coastal region of Korea. Using whole genome sequencing (WGS) and advanced bioinformatics tools, we identified novel sequence types through multilocus sequence typing and serotyped isolates using the VPsero database. Pathogenic genes, such as *tdh* and *trh*, were detected in only a few isolates, suggesting the involvement of alternative virulence mechanisms in the pathogenicity of these strains. An in silico analysis revealed widespread AMR, particularly against beta-lactams, chloramphenicol, and tetracycline antibiotics, underscoring the public health threats posed by these strains. A phylogenetic analysis revealed no significant clustering by geographic origin, year, or strain source, although most clinical and environmental strains were not closely related at lower phylogenetic branches. These findings highlight the importance of continued genomic surveillance and strict regulations regarding antibiotic use in marine environments. Moreover, this study suggests that integrating WGS data with epidemiological models could enhance the prediction of the emerging virulent strains and support effective outbreak management strategies.

## 1. Introduction

*Vibrio parahaemolyticus* is a halophilic Gram-negative bacterium commonly found in marine environments that is transmitted to humans through the consumption of contaminated seafood. It is recognized as a major cause of gastroenteritis, and in severe cases, septicemia [1,2,3]. This pathogen has been reported globally, including in regions such as North America [4,5], South America [6], Europe [7], and Asia [8,9]. The ongoing increase in infections is likely exacerbated by ocean temperature increases, driven by global climate change [10]. In aquaculture, *V. parahaemolyticus* also poses a substantial economic burden by causing vibriosis, which leads to high mortality rates in farmed aquatic species [11,12]. Despite the widespread impact of this pathogen, its virulence factors remain poorly understood. Thermostable direct hemolysin (*tdh*) and thermostable-related hemolysin (*trh*) genes are often used as key molecular markers for pathogenic strains [13,14]. However, recent studies have shown pathogenicity in strains lacking these genes, implicating additional virulence factors such as *toxR* and Type III secretion systems in mediating pathogenic mechanisms [15,16,17,18]. The emergence of antibiotic-resistant *V. parahaemolyticus* strains presents an even greater public health threat, driven by the overuse of antibiotics in aquaculture and clinical settings [19,20]. This necessitates the development of robust genomic surveillance strategies to mitigate the risks associated with these multidrug-resistant strains.

Traditionally, multilocus sequence typing (MLST), which targets seven housekeeping genes (*recA*, *gyrB*, *dnaE*, *dtdS*, *pyrC*, *pntA*, and *tnaA*), is the method of choice for strain differentiation [21]. Although MLST provides a straightforward approach for phylogenetic classification, its resolution is limited because of the small number of loci analyzed. Whole genome sequencing (WGS) offers a comprehensive and high-resolution approach that enables the identification of genetic variants and provides deeper insights into the evolutionary and pathogenic traits of *V. parahaemolyticus*.

Considering that there are many countries with high seafood consumption, including South Korea, it is crucial to conduct continuous monitoring and perform detailed genomic characterization of *Vibrio parahaemolyticus* using whole genome sequencing (WGS) data. Such efforts are essential to detect and understand the genetic diversity and potential risks posed by this pathogen in regions where seafood is widely consumed. Accordingly, this study was designed to produce and examine high-quality WGS data, specifically for *V. parahaemolyticus* strains collected from the southern coastal areas of Korea, providing a foundation for ongoing surveillance and characterization efforts.

## 2. Materials and Methods

### 2.1. Sample Isolation and DNA Extraction

The *V. parahaemolyticus* isolates used in this study were collected from seawater and shellfish (oysters, clams, and ark shells) along the southern coast of Korea between 2019 and 2023. Shellfish meat (200 g) was homogenized with 200 mL phosphate-buffered solution (PBS; 2.5 mM KH_2_PO_4_; pH 7.2). The homogenates were inoculated into 10 mL alkaline peptone water (APW; 2% NaCl, pH 8.5 ± 0.2) and incubated at 37 °C for 18–24 h. Seawater samples were treated similarly by inoculation into APW and incubation under the same conditions. After incubation, 10 μL of each of the positive APW cultures (both shellfish and seawater samples) was streaked onto CHROMagar™ Vibrio plates (CHROMagar, La Plaine St-Denis, France) and incubated at 37 °C for 24 h. Purple colonies were presumptively identified as *V. parahaemolyticus*, and approximately 3–5 colonies were selected for further screening through oxidase reaction and fermentation activity on triple-sugar iron agar. Final confirmation of *V. parahaemolyticus* was conducted using matrix-assisted laser desorption ionization–time of flight mass spectrometry (Bruker Daltonics, Bremen, Germany). Genomic DNA was extracted using the Bacterial Genomic DNA Extraction Kit Ver. 3.0 (Takara, Shiga, Japan).

### 2.2. Whole Genome Sequencing, Contig Assembly, and Annotation

Draft genome sequencing of *V. parahaemolyticus* was performed using an Illumina MiSeq platform (Illumina, San Diego, CA, USA). Library preparation was performed according to the manufacturer’s protocol using an Illumina DNA Prep Kit (Illumina, San Diego, CA, USA). Sequencing was conducted by paired-end sequencing (2 × 300 bp) for 600 cycles on an Illumina MiSeq system using the MiSeq Reagent Kit v3. Raw reads were trimmed using BBduk v38.96 (k = 23, hdist = 1, minlen = 30, qtrim = r, trimq = 15, and ziplevel = 4). Genomic sequence contigs were de novo assembled using Shovill v1.1.0. Draft genomes were annotated using Prokka v1.14.6 software [22]. Additionally, we obtained the type information of the O and K antigens using VPsero v1.0.0 software to obtain the serotype of *V. parahaemolyticus* [23].

### 2.3. Reference Data Download

In this study, 127 *V. parahaemolyticus* genome datasets with clear metadata (source, nation, and sampling year) and assembly levels higher than those of the chromosome were obtained from the National Center for Biotechnology Information (NCBI) database (Table S1). Samples obtained from human specimens with confirmed symptoms of *V. parahaemolyticus* pathogenicity were marked as clinical, whereas the other samples were marked as environmental and foodborne.

### 2.4. Pangenome Analysis

To obtain comprehensive core gene information across all strains, we conducted a pan-genome analysis using Panaroo v1.3.0 software, applying a stringent threshold of 80% identity and coverage. This threshold ensured that only genes present in all strains were identified as core genes, thereby enhancing the reliability of the analysis for understanding conserved genetic elements within the dataset [24]. Subsequently, the amino acid sequences obtained from the core genes were meticulously aligned using the advanced multiple sequence alignment capabilities of MAFFT v7.310, which provided a robust framework for accurate alignment across diverse sequences [25].

### 2.5. Maximum Likelihood Phylogenetic Analysis

Two types of phylogenetic analyses were performed using IQ-Tree v2.0.3 software [26]. The first analysis used amino acid alignment of the total core genes to identify the precise genetic clades of *V. parahaemolyticus*. The second method used MLST with only seven housekeeping gene alignments (*dnaE*, *gyrB*, *recA*, *dtdS*, *pntA*, *pyrC*, and *tnaA*) to obtain the summarized phylogenetic information. Each analysis was estimated using the maximum likelihood method, and the results were visualized using Interactive Tree of Life (iTOL) v6.9.1 software [27].

### 2.6. In Silico MLST and AMR Genes

To acquire multilocus sequence typing (MLST) data for *Vibrio parahaemolyticus*, the sequence information from seven essential housekeeping genes (*dnaE*, *gyrB*, *recA*, *dtdS*, *pntA*, *pyrC*, and *tnaA*) was analyzed using mlst v2.11 software. These data were cross-referenced with the PubMLST database (https://pubmlst.org/, accessed on 21 August 2024) to determine the specific sequence type (ST) associated with each sample [28]. Additionally, the identification of antimicrobial resistance (AMR) genes within *V. parahaemolyticus* strains was performed through AMRFinderPlus v3.11 software, utilizing the NCBI Bacterial Antimicrobial Resistance Reference Gene Database to confirm the presence of resistance genes [29].

## 3. Results

### 3.1. Genome Assembly and Pangenome Analysis

De novo assembly was performed on 32 *V. parahaemolyticus* isolates collected from environmental and foodborne sources in the southern seas of Korea between 2019 and 2023. The average read sequence was 605 Mb with an average mapping depth of x117 ± 26.67. The average size of the assembled genome was 5.22 Mb, and the average guanine-cytosine (GC) content was 45.44%, which is similar to the NCBI reference sequence. Detailed results are presented in Table 1. A pangenome analysis was conducted on the assembled genome along with 127 datasets provided by NCBI. The genome comprised 22,566 protein-coding genes with an average of 4549.76 genes per sample. A total of 2812 core genes were identified in all samples.

### 3.2. Serotyping Analysis and MLST

In this study, eight unique sequence types (STs) were identified among the 32 *V. parahaemolyticus* strains isolated from the southern coastal waters of Korea. Interestingly, most of these strains represented novel STs that are not currently cataloged in the pubMLST database, highlighting the unique genetic characteristics of Korean isolates. In contrast, an analysis of clinical strains from the NCBI database revealed nine STs (3, 34, 36, 110, 162, 224, 332, 631, and 799) that were exclusively found in clinical samples, with none of these STs being present in environmental or foodborne strains.

A serotype analysis conducted with the VPsero database revealed that the K serotype could not be determined for the majority of the isolates. In contrast, the O serotype was successfully identified in all isolates except for five specific samples, indicating a higher success rate in identifying O serotypes among the strains analyzed. Among the nine O serotypes detected, O11 was the most frequently observed, accounting for 28.13% of the samples, followed by O5 at 18.75%. This suggests that these serotypes may be more prevalent in the local strain population. However, despite the overall success in identifying O serotypes, certain challenges remained in fully characterizing the unassigned samples. To address this, we expanded our analysis beyond the three target genes used in the VPsero method to include broader related genomic regions. Nevertheless, even with this extended approach, these samples still did not align with any previously known K serotypes (Table S2; Figures S3 and S4).

Additionally, sequence information was obtained for five key genes related to pathogenicity: *tdh*, *trh*, *tlh*, *toxR*, and *toxS*. Among these, three genes—*tlh*, *toxR*, and *toxS*—were consistently confirmed in all tested strains, underscoring their presence across the board. In contrast, the tdh gene was detected in only two of the samples (6.25%), while the *trh* gene was identified in four samples (12.5%). A comparison with clinical data acquired from the NCBI database showed similar findings, where *tlh*, *toxR*, and *toxS* were confirmed in all clinical strains, as was observed in the Korean strains. However, in clinical samples, *tdh* was present in 15 samples (42.86%), and *trh* was observed in eight samples (22.86%), indicating a higher detection rate of these genes in clinical strains compared to the local isolates.

### 3.3. In Silico AMR Genes

The potential risk of *V. parahaemolyticus* infection was identified using an in silico AMR gene presence/absence analysis (Figure S1). Fifty AMR genes consisting of 10 antibiotic subclasses were identified. Most samples showed strong resistance to β-lactams, chloramphenicol, and tetracycline antibiotics. Interestingly, 157 strains contained the type C chloramphenicol O-acetyltransferase (*catC*) gene (98.74%), and 158 and 157 strains contained the oxytetracycline resistance phosphoribosyltransferase domain-containing protein Tet(34) [*tet(34)*; 99.37%] and the tetracycline efflux Na+/H+ antiporter family transporter Tet(35) [*tet(35)*; 98.11%] genes, respectively, indicating that specific gene families of the chloramphenicol and tetracycline subclasses were highly conserved. Although less frequent than reported previously, the carbenicillin-hydrolyzing class A beta-lactamase CARB-18 (*blaCARB-18*) gene was also dominant in the beta-lactam subclass (67.92%), and it is included in most Korean strains (95.45%). These results highlight the utility of genomic data in predicting antibiotic resistance genes in *V. parahaemolyticus*. However, further experimental validation is necessary to confirm the functional expression and phenotypic impact of these resistance genes.

### 3.4. Whole-Genome and MLST Phylogenic Analyses

**Figure 1:**
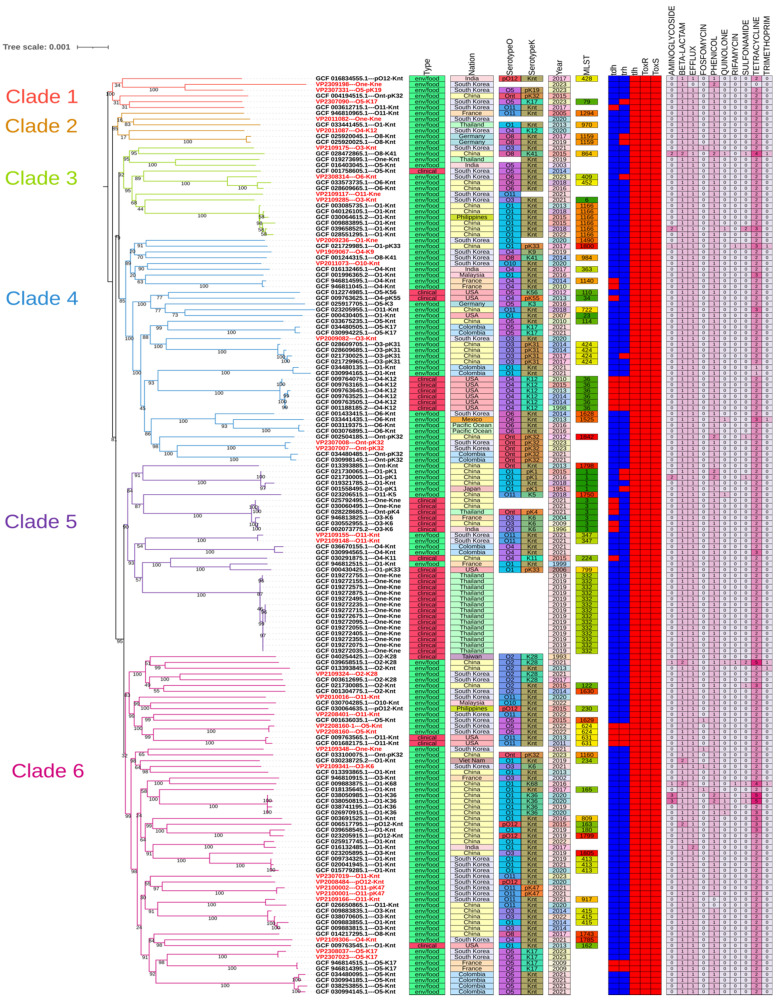
Whole-genome phylogenetic analysis of 32 *Vibrio parahaemolyticus* isolates from southern seas of Korea, including 127 reference data points. Samples obtained from human specimens with confirmed symptoms were labeled as clinical in “Type” layer, and other samples were labeled as environmental and foodborne. “Virulence” layer (*tdh*, *trh*, *tlh*, *toxR*, and *toxS*) shows presence–absence pathogenicity-related genes. Presence is color-coded; red indicates presence of virulence factors, while no color indicates their absence. Last layer shows number of antimicrobial resistance genes. Abbreviations: MLST, multilocus sequence typing; env, environment.

A comprehensive phylogenetic analysis based on whole-genome data was conducted using a dataset of 127 *V. parahaemolyticus* genomes collected from 15 different countries, all obtained from the NCBI database. This analysis centered on a set of 2812 core genes shared across the genomes to construct a robust phylogenetic tree. The resulting tree highlighted six genetically distinct clades, but without discernible patterns tied to either the geographic origins of the isolates or their respective years of sampling (Figure 1). Interestingly, the 32 isolates originating from Korea were spread across all six clades, showing no notable clustering or exclusive grouping by region. Furthermore, most clinical isolates did not group closely with environmental or foodborne strains, indicating possible genetic divergence between strains based on origin or source. One notable exception was isolate VP2109306, which shared a close sub-branch with the environmental isolate GCF_009763545.1, suggesting a rare overlap.

An MLST-based phylogenetic analysis was conducted on 116 genomes containing seven housekeeping genes (*dnaE*, *gyrB*, *recA*, *dtdS*, *pntA*, *pyrC*, and *tnaA*; Figure S2). Although the clustering pattern observed from the ST data was consistent with the whole-genome analysis, the resolution was significantly lower, making it difficult to distinguish between clades in fine detail.

## 4. Discussion

This study presents an in-depth genomic analysis of *V. parahaemolyticus* isolates from environmental and foodborne sources in the southern sea of Korea, providing valuable insights into the genetic structure of the pathogen and its public health implications. Using in silico MLST and serotyping, we identified several novel STs that have not been registered in existing databases. These findings reflect the genetic uniqueness of the Korean isolates and align with the hypothesis that rising sea temperatures driven by global climate change may increase the incidence and genetic diversity of *V. parahaemolyticus* in marine environments. Furthermore, the unassigned samples identified in this study may represent novel serotypes that are yet to be characterized, emphasizing the need for more comprehensive data and deeper investigations. As additional datasets become available, further research should focus on refining serotype classifications and uncovering the broader bacterial diversity, which is critical for improving our understanding of the ecological and pathogenic potential of this species.

Regarding virulence, three pathogenic genes (*tlh*, *toxR*, and *toxS*) were found in all isolates, whereas two primary pathogenic genes (*tdh* and *trh*) were present in only a few strains. This could be considered a positive finding for public health as it suggests that most of the isolates lacked these major virulence factors; however, the limitation is that research results confirming pathogenicity without these genes have been continuously reported [16,17,30,31]. A phylogenetic analysis revealed that most clinical strains did not share subbranches with environmental or foodborne strains. However, the pathogenic GCF_009763545.1 and VP2109306 strains isolated from the southern sea of Korea shared a sub-branch, indicating a close genetic relationship. Considering that environment/foodborne strains have never been confirmed to have symptoms under clinical conditions but still possess the potential for pathogenicity, their latent risks should not be overlooked. Additionally, because they do not carry *tdh* and *trh*, they are difficult to detect using conventional polymerase chain reaction methods. Although this study primarily provides genomic insights, understanding the functional relevance of these identified pathogenic genes will require complementary transcriptomic or proteomic analyses. Such approaches can elucidate gene expression profiles under different environmental or host–pathogen interaction conditions, offering a more comprehensive understanding of *V. parahaemolyticus* pathogenicity and its underlying mechanisms.

An MLST-based phylogenetic analysis also has limitations in accurately describing the relationships between strains because of its low resolution. A phylogenetic analysis of whole genomes is expected to overcome these limitations by providing more precise genetic relationships. Although these results suggest a lower immediate health risk for these isolates, the continued monitoring and investigation of alternative pathogenic pathways are crucial.

An in silico AMR analysis revealed that most isolates carried multiple resistance genes, particularly against beta-lactams, chloramphenicol, and tetracycline antibiotics. These results show that most strains, including those from Korea, had an average of more than four AMR genes. Previous studies using antibiotic susceptibility tests have reported resistance only to beta-lactam antibiotics, including ampicillin, amoxicillin, cefazolin, and penicillin [32,33,34,35,36]. The presence of additional AMR genes indicates a broader spectrum of resistance than previously thought, which is potentially driven by environmental pressures such as the overuse of antibiotics in aquaculture. This raises significant public health concerns, particularly in light of studies suggesting that AMR genes can be transferred across geographical regions through various environmental channels, including water currents, shipping, and the seafood trade [37]. These findings provide a comprehensive genomic basis for understanding the prevalence of AMR genes in *V. parahaemolyticus*. However, the lack of experimental validation represents a limitation of this study. While genomic data are invaluable for predicting resistance profiles, experimental validation is necessary to confirm the functional implications and phenotypic impact of these AMR genes. Future research should focus on integrating these experimental approaches to strengthen the reliability of genomic predictions and to better understand their relevance in real-world conditions.

This study offers valuable insights into the genetic structure and public health implications of *V. parahaemolyticus* isolates from the southern sea of Korea. Nevertheless, several limitations must be acknowledged. First, the use of in silico methods for MLST, serotyping, and antibiotic resistance gene analysis has inherent limitations as the results may not always align with phenotypic analyses or actual gene expression data. To address this, future research should incorporate experimental validation methods, such as Minimum Inhibitory Concentration assays, to confirm antibiotic resistance profiles and protein expression studies to characterize the functional relevance of pathogenic genes. These approaches can bridge the gap between genomic predictions and practical biological applications. Additionally, this study primarily focuses on environmental and foodborne isolates, with limited data available for clinical strains. Although relationships between certain clinical strains and the isolates were observed, a more comprehensive investigation involving a larger number of clinical isolates is necessary to better understand the potential transmission pathways and health risks. Addressing these limitations in future research is essential for gaining a more accurate understanding of *V. parahaemolyticus* pathogenicity and resistance mechanisms.

In conclusion, the findings of this study emphasize the need for continued genomic surveillance of *V. parahaemolyticus* populations in marine environments, particularly in coastal regions, such as Korea, where seafood consumption is high. The discovery of novel STs and the widespread presence of AMR genes in these isolates suggest that ongoing monitoring is essential to anticipate and mitigate potential public health risks. Future research should focus on expanding the geographical range of the isolates and incorporating comparative genomic approaches to identify key evolutionary drivers of both pathogenicity and AMR. Additionally, as WGS continues to improve, integrating these data into broader epidemiological models will be critical for predicting the emergence of new virulent strains and for managing potential outbreaks.

## 5. Conclusions

This study advances the understanding of the public health risks associated with *Vibrio parahaemolyticus* in Korea, a region vulnerable to climate-driven increases in ocean temperature. This study provides a comprehensive whole-genome analysis of strains isolated from the southern coast of Korea, moving beyond partial genetic studies to assess their genetic diversity, virulence potential, and antimicrobial resistance (AMR). The findings reveal diverse genetic profiles with novel sequence types and highlight significant AMR against beta-lactams, chloramphenicol, and tetracycline antibiotics. This study identified the core pathogenic genes, *tdh* and *trh*, in a few strains, suggesting alternate virulence mechanisms among different strains. Furthermore, the absence of dominant genetic clusters across geographic locations emphasizes the unpredictable nature of strain emergence and spread. This study holds broad utility for public health, emphasizing the need for continuous genomic surveillance of marine pathogens and strict regulation of antibiotic use in aquatic environments. The integration of genomic data with epidemiological models can improve outbreak prediction and management strategies, contributing to national and global efforts to control infections. The findings of this study may serve as a critical foundation for future research, offering insights essential for protecting public health in coastal regions with high seafood consumption.

## Figures and Tables

**Table 1:** Whole-genome de novo assembly states of 32 *Vibrio parahaemolyticus* isolates from southern seas of Korea.

ID	No. of Contigs	Contigs (bp)	Gap (%)	Contigs N50	Contigs L50	Contigs N90	Contigs L90	Max Contigs (bp)	Avg. GC	Std. GC
VP1909067	79	5,232,868	0	3	800,967	10	101,040	1,069,668	0.4526	0.06193
VP2008484	87	5,132,100	0	4	553,633	12	72,881	874,128	0.45249	0.05309
VP2009082	78	4,974,122	0	3	518,497	11	100,891	1,312,245	0.45444	0.05463
VP2009236	96	5,402,922	0	5	327,364	16	86,352	945,765	0.45065	0.04817
VP2010016	82	5,364,697	0	3	748,205	8	100,153	1,759,386	0.45139	0.05903
VP2011073	59	5,038,997	0	3	535,647	8	100,880	1,310,199	0.453	0.05864
VP2011082	93	5,035,923	0	3	611,085	13	81,815	1,331,558	0.45318	0.049
VP2011087	95	5,056,646	0	4	494,667	16	82,468	944,451	0.45312	0.05099
VP2100001	69	5,234,396	0	4	579,397	9	123,331	870,412	0.4522	0.04376
VP2100002	75	5,247,989	0	4	621,515	10	100,605	822,563	0.4523	0.04433
VP2109117	95	5,361,784	0	5	415,944	15	82,202	1,146,534	0.45175	0.04339
VP2109148	116	5,347,755	0	4	457,876	13	101,038	833,642	0.45175	0.05549
VP2109155	109	5,347,413	0	5	443,583	14	81,648	814,009	0.45176	0.05182
VP2109166	103	5,233,806	0	4	434,018	14	90,324	1,399,639	0.45273	0.06232
VP2109175	141	5,098,948	0	3	523,293	10	112,377	1,776,237	0.45317	0.05648
VP2109285	126	5,093,053	0	2	720,522	11	100,025	1,837,928	0.45297	0.06521
VP2109306	87	5,206,279	0	3	796,362	12	89,427	975,214	0.4525	0.0536
VP2109324	90	5,057,999	0	3	574,000	10	72,599	1,359,917	0.45268	0.05466
VP2109341	130	5,238,815	0	5	465,158	15	72,880	753,850	0.4516	0.05336
VP2109348	74	5,040,387	0	3	643,478	10	122,181	1,352,678	0.45303	0.05259
VP2208160-1	83	5,142,854	0	3	787,080	7	122,766	1,272,847	0.45299	0.06302
VP2208160	83	5,143,047	0	3	787,080	7	122,611	1,272,847	0.45299	0.06012
VP2208401	51	5,234,969	0	2	1,201,182	7	175,093	1,417,288	0.4519	0.0558
VP2307007	64	5,151,186	0	4	393,880	14	111,876	1,274,736	0.4522	0.04839
VP2307008	69	5,151,931	0	4	393,880	14	111,876	1,274,736	0.4522	0.05518
VP2307019	66	5,174,459	0	3	527,678	9	122,910	1,323,216	0.45147	0.05157
VP2307023	87	5,253,567	0	4	527,770	13	84,107	1,302,206	0.45257	0.04273
VP2307090	107	5,263,459	0	6	347,169	18	83,137	539,048	0.4509	0.05168
VP2307331	63	5,363,343	0	2	933,557	7	100,772	1,878,832	0.45125	0.05732
VP2308037	337	5,635,722	0	4	527,604	18	50,349	1,302,201	0.45487	0.07508
VP2308314	104	5,956,752	0	4	483,066	15	89,008	1,737,544	0.44043	0.0607
VP2309198	10,797	10,460,359	0	611	3296	6,826	315	109,757	0.453	0.03836
VP1909067	79	5,232,868	0	3	800,967	10	101,040	1,069,668	0.4526	0.06193
VP2008484	87	5,132,100	0	4	553,633	12	72,881	874,128	0.45249	0.05309
VP2009082	78	4,974,122	0	3	518,497	11	100,891	1,312,245	0.45444	0.05463
VP2009236	96	5,402,922	0	5	327,364	16	86,352	945,765	0.45065	0.04817
VP2010016	82	5,364,697	0	3	748,205	8	100,153	1,759,386	0.45139	0.05903
VP2011073	59	5,038,997	0	3	535,647	8	100,880	1,310,199	0.453	0.05864
VP2011082	93	5,035,923	0	3	611,085	13	81,815	1,331,558	0.45318	0.049
VP2011087	95	5,056,646	0	4	494,667	16	82,468	944,451	0.45312	0.05099
VP2100001	69	5,234,396	0	4	579,397	9	123,331	870,412	0.4522	0.04376
VP2100002	75	5,247,989	0	4	621,515	10	100,605	822,563	0.4523	0.04433

## Data Availability

All sequencing data have been deposited into the National Center for Biotechnology Information (NCBI) sequence read archive (SRA) database under the accession number SRX26397254-SRX26397285.

## Supplementary Materials

The following supporting information can be downloaded at https://www.mdpi.com/article/10.3390/microorganisms12122497/s1, Figure S1: Presence of antimicrobial resistance (AMR) genes test based on NCBI Bacterial Antimicrobial Resistance Reference Gene Database; Figure S2: Multilocus sequence typing (MLST) phylogenetic analysis of 32 *Vibrio parahaemolyticus* isolates from southern seas of Korea, including 116 reference data; Figure S3: Comparative analysis of gene clusters in identified O-loci; Figure S4: Comparative analysis of gene clusters in identified K-loci; Table S1: Sample metadata for NCBI reference data table; Table S2. Results of serotype analysis using alternative serotype-related loci sequence-based method.
