# Supplementary material for: Genomic Insights into Vibrio parahaemolyticus from Southern Korea: Pathogenicity, Antimicrobial Resistance, and Phylogenetic Distinctions
Source: Microorganisms. 2024 Dec 4;12(12):2497. doi: 10.3390/microorganisms12122497 (PMC11727765; doi:10.3390/microorganisms12122497)
Supplement: Supplementary file 1 [file microorganisms-12-02497-s001.zip › microorganisms-3335565-supplementary.pdf]

## Supplementary Information

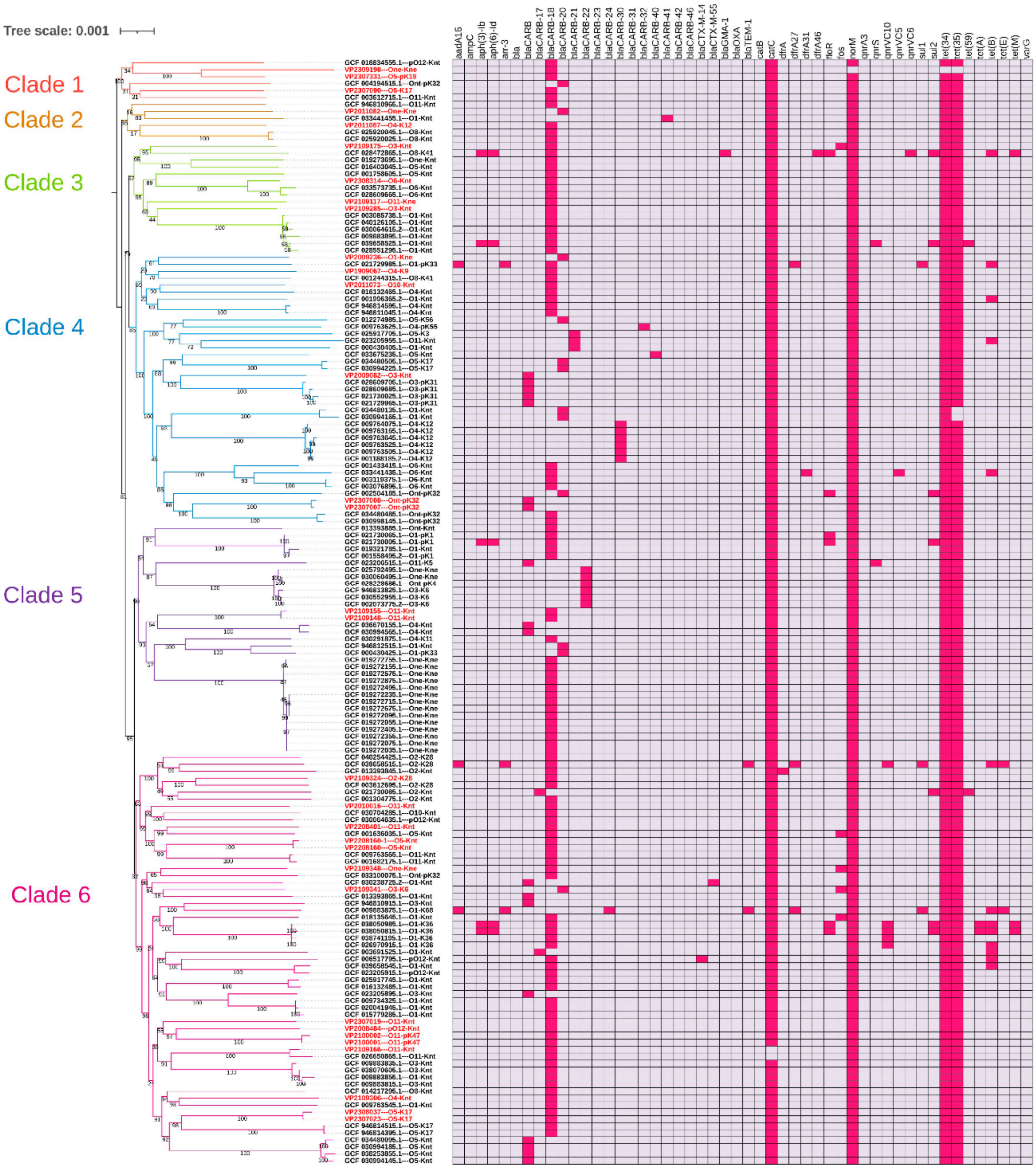


**Figure S1.** Presence of antimicrobial resistance (AMR) genes test based on NCBI Bacterial Antimicrobial Resistance Reference Gene Database. Presence is color-coded: red indicates the presence of the virulence factors, while no color indicates their absence.

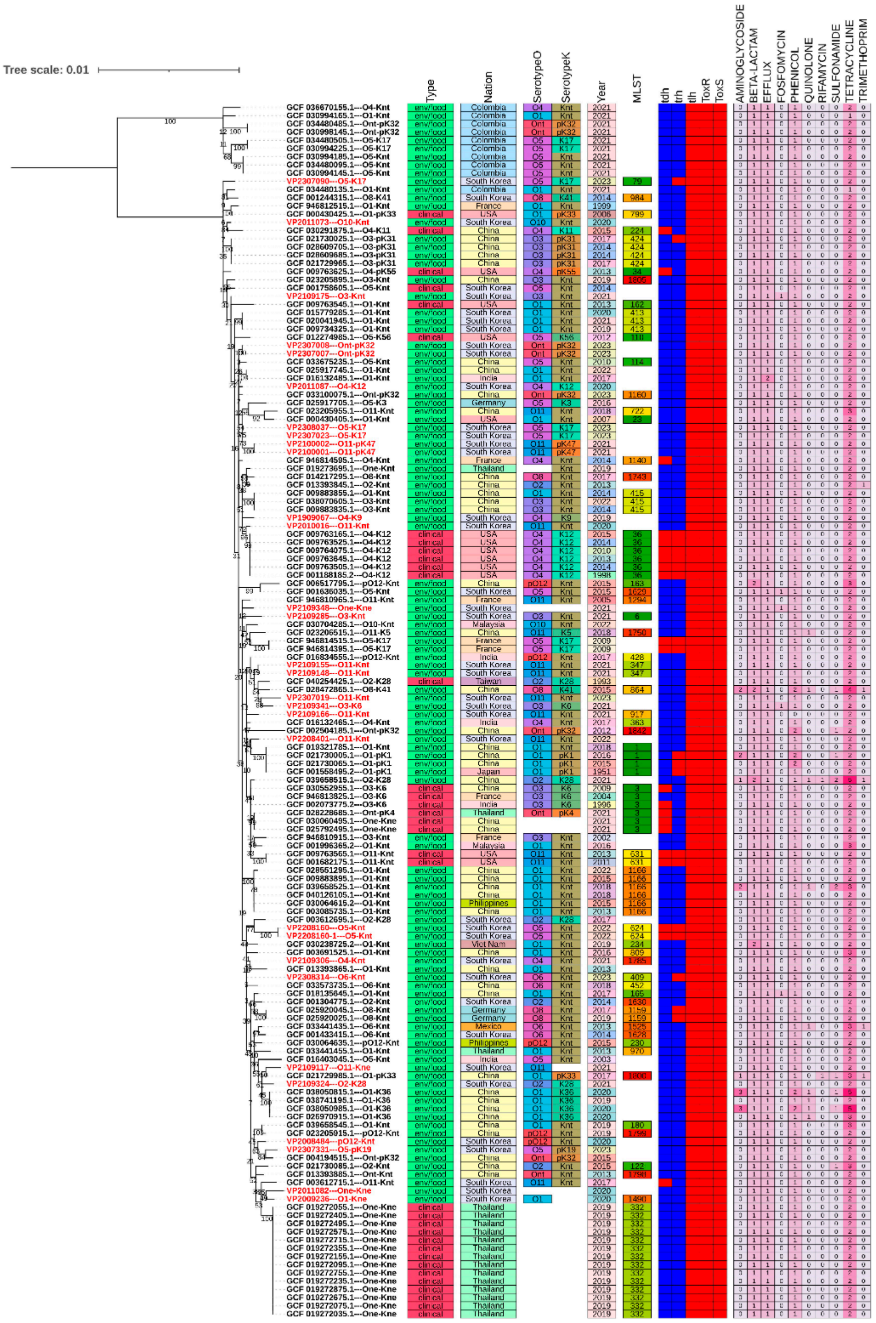

**Figure S2.** Multilocus sequence typing (MLST) phylogenetic analysis of 32 Vibrio parahaemolyticus isolates from the southern seas of Korea, including 116 reference data. Samples obtained from human specimens with confirmed symptoms were labeled as clinical in the "Type" layer, and other samples were labeled as environmental & foodborne. The "Virulence" layer (*tdh, trh, tlh, toxR, toxS*) shows the presence-absence of pathogenicity-related genes. Presence is color-coded: red indicates the presence of the virulence factors, while no color indicates their absence. The last layer shows the number of antimicrobial resistance genes present.

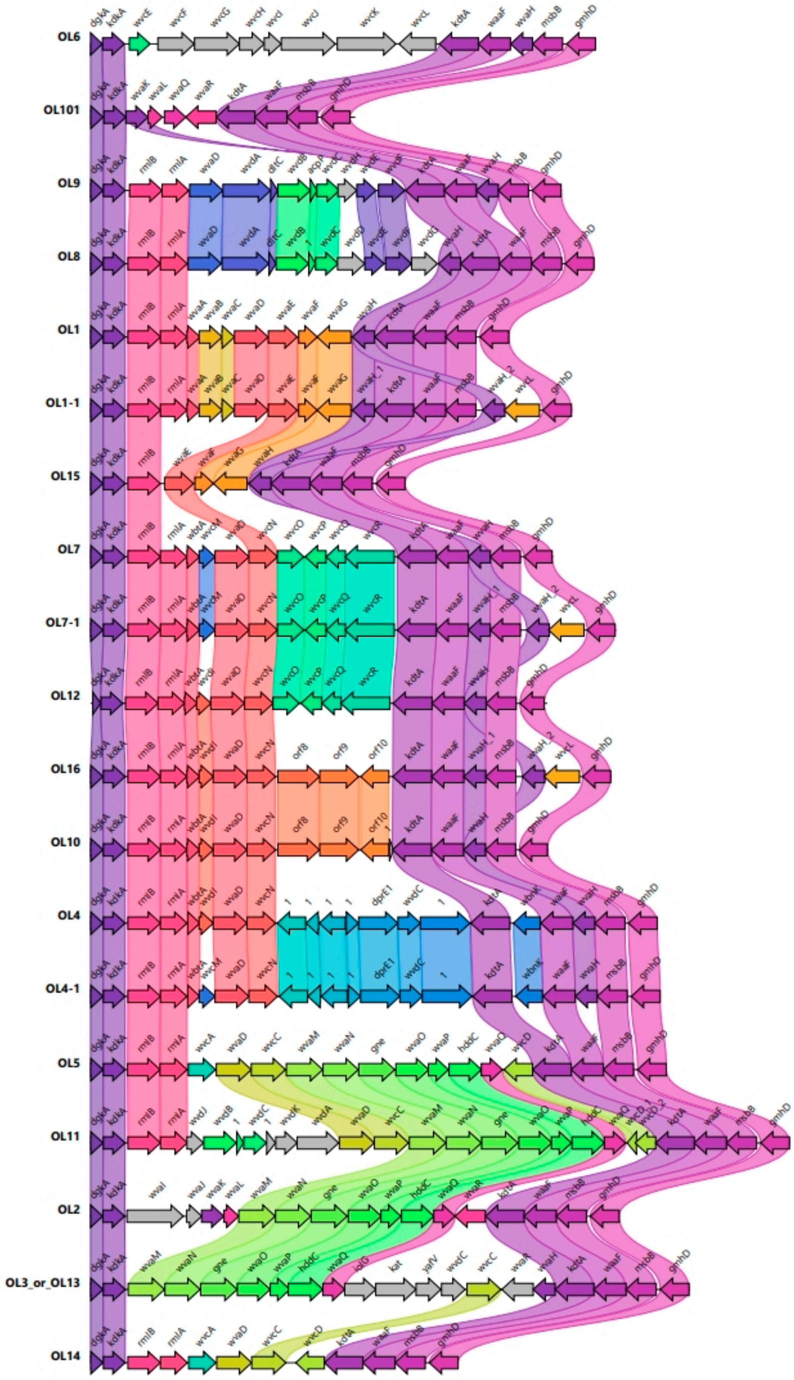


**Figure S3.** Gene cluster comparisons of the identified O-loci. All similar gene homologs have been assigned an unique color and are linked with similar color boxes. Hypothetical proteins are assigned with an "1".

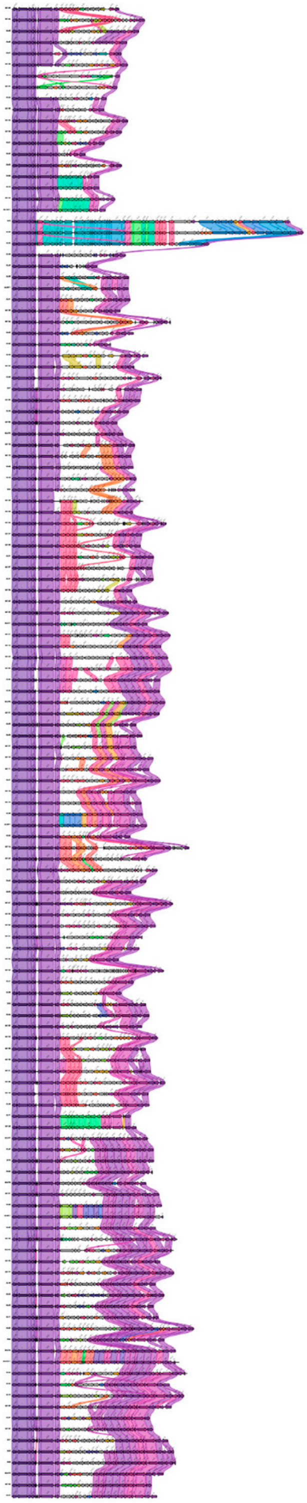

**Figure S4.** Gene cluster comparisons of the identified K-loci. All similar gene homologs have been assigned an unique color and are linked with similar color boxes. Hypothetical proteins are assigned with an "1".

**Table S1.** Sample metadata for NCBI reference data table.

| NCBI RefSeq ID | year | nation | source | host | type | AssemblyLevel | mlst | serotype |
|---|---|---|---|---|---|---|---|---|
| GCF_000430405.1 | 2007 | USA | oyster | | env/food | Complete Genome | 23 | O1:Knt |
| GCF_000430425.1 | 2006 | USA | stool | *Homo sapiens* | clinical | Complete Genome | 799 | O1:pK33 |
| GCF_001188185.2 | 1998 | USA | stool | *Homo sapiens* | clinical | Complete Genome | 36 | O4:K12 |
| GCF_001244315.1 | 2014 | South Korea | | Finespotted flounder | env/food | Complete Genome | 984 | O8:K41 |
| GCF_001304775.1 | 2014 | South Korea | cutting board | | env/food | Complete Genome | 1630 | O2:Knt |
| GCF_001433415.1 | 2014 | South Korea | aquarium water | | env/food | Complete Genome | 1628 | O6:Knt |
| GCF_001558495.2 | 1951 | Japan | shirasu | Environment | env/food | Complete Genome | 1 | O1:pK1 |
| GCF_001636035.1 | 2015 | South Korea | | toothfish | env/food | Complete Genome | 1629 | O5:Knt |
| GCF_001682175.1 | 2011 | USA | stool | N | clinical | Complete Genome | 631 | O11:Knt |
| GCF_001758605.1 | 2014 | South Korea | patient | *Homo sapiens* | clinical | Complete Genome | - | O5:Knt |
| GCF_001996365.2 | 2016 | Malaysia | shrimp pond | | env/food | Complete Genome | - | O1:Knt |
| GCF_002073775.2 | 1996 | India | human | *Homo sapiens* | clinical | Complete Genome | 3 | O3:K6 |
| GCF_002504185.1 | 2012 | China | Aquaculture environment | | env/food | Chromosome | 1842 | Ont:pK32 |
| GCF_003076895.1 | 2016 | Pacific Ocean | | *Penaeus vannamei* | env/food | Complete Genome | - | O6:Knt |
| GCF_003085735.1 | 2013 | China | | *Penaeus vannamei* | env/food | Complete Genome | 1166 | O1:Knt |
| GCF_003119375.1 | 2016 | Pacific Ocean | | *Penaeus vannamei* | env/food | Complete Genome | - | O6:Knt |
| GCF_003612695.1 | 2017 | South Korea | Unknown | | env/food | Complete Genome | - | O2:K28 |
| GCF_003612715.1 | 2017 | South Korea | Unknown | | env/food | Complete Genome | - | O11:Knt |
| GCF_003691525.1 | 2016 | China | shrimp | *Penaeus vannamei* | env/food | Complete Genome | 809 | O1:Knt |
| GCF_004194515.1 | 2015 | China | seawater | | env/food | Complete Genome | - | Ont:pK32 |
| GCF_006517795.1 | 2015 | China | market | | env/food | Complete Genome | 163 | pO12:Knt |
| GCF_009734325.1 | 2019 | South Korea | hepatopancreas | *Penaeus vannamei* | env/food | Complete Genome | 413 | O1:Knt |
| GCF_009763165.1 | 2015 | USA | stool | | clinical | Complete Genome | 36 | O4:K12 |
| GCF_009763505.1 | 2014 | USA | stool | | clinical | Complete Genome | 36 | O4:K12 |
| GCF_009763525.1 | 2014 | USA | stool | | clinical | Complete Genome | 36 | O4:K12 |
| GCF_009763545.1 | 2013 | USA | oral swab | | clinical | Complete Genome | 162 | O1:Knt |
| GCF_009763565.1 | 2013 | USA | stool | | clinical | Complete Genome | 631 | O11:Knt |
| GCF_009763625.1 | 2013 | USA | stool | | clinical | Complete Genome | 34 | O4:pK55 |
| GCF_009763645.1 | 2013 | USA | stool | | clinical | Complete Genome | 36 | O4:K12 |

| NCBI RefSeq ID | year | nation | source | host | type | AssemblyLevel | mlst | serotype |
|---|---|---|---|---|---|---|---|---|
| GCF_009764075.1 | 2010 | USA | stool | | clinical | Complete Genome | 36 | O4:K12 |
| GCF_009883815.1 | 2014 | China | AHPND-infected *Litopenaeus vannamei* | *Penaeus vannamei* | env/food | Complete Genome | - | O3:Knt |
| GCF_009883835.1 | 2014 | China | AHPND-infected *Penaeus japonicus* | *Penaeus japonicus* | env/food | Complete Genome | 415 | O3:Knt |
| GCF_009883855.1 | 2014 | China | AHPND-infected *Litopenaeus vannamei* | *Penaeus vannamei* | env/food | Complete Genome | 415 | O1:Knt |
| GCF_009883875.1 | 2016 | China | AHPND-infected *Litopenaeus vannamei* | *Penaeus vannamei* | env/food | Complete Genome | - | O1:K68 |
| GCF_009883895.1 | 2015 | China | AHPND-infected *Penaeus japonicus* | *Penaeus japonicus* | env/food | Complete Genome | 1166 | O1:Knt |
| GCF_012274985.1 | 2012 | USA | stool | | clinical | Chromosome | 110 | O5:K56 |
| GCF_013393845.1 | 2013 | China | crayfish | | env/food | Complete Genome | - | O2:Knt |
| GCF_013393865.1 | 2013 | China | crayfish | | env/food | Complete Genome | - | O1:Knt |
| GCF_013393885.1 | 2013 | China | crayfish | | env/food | Complete Genome | 1798 | Ont:Knt |
| GCF_014217295.1 | 2017 | China | hepatopancreas | *Penaeus vannamei* | env/food | Complete Genome | 1743 | O8:Knt |
| GCF_015779285.1 | 2020 | South Korea | | *Penaeus vannamei* | env/food | Complete Genome | 413 | O1:Knt |
| GCF_016132465.1 | 2017 | India | diseased shrimp | | env/food | Chromosome | 363 | O4:Knt |
| GCF_016132485.1 | 2017 | India | diseased shrimp | | env/food | Chromosome | - | O1:Knt |
| GCF_016403045.1 | 2003 | India | seafood | | env/food | Chromosome | - | O5:Knt |
| GCF_016834555.1 | 2017 | India | diseased shrimp | | env/food | Chromosome | 428 | pO12:Knt |
| GCF_018135645.1 | 2017 | China | | *Penaeus vannamei* | env/food | Complete Genome | 165 | O1:Knt |
| GCF_019272035.1 | 2019 | Thailand | rectal swab | | clinical | Chromosome | 332 | One:Kne |
| GCF_019272055.1 | 2019 | Thailand | rectal swab | | clinical | Chromosome | 332 | One:Kne |
| GCF_019272075.1 | 2019 | Thailand | rectal swab | | clinical | Chromosome | 332 | One:Kne |
| GCF_019272095.1 | 2019 | Thailand | rectal swab | | clinical | Chromosome | 332 | One:Kne |
| GCF_019272155.1 | 2019 | Thailand | rectal swab | | clinical | Chromosome | 332 | One:Kne |
| GCF_019272235.1 | 2019 | Thailand | rectal swab | | clinical | Chromosome | 332 | One:Kne |
| GCF_019272355.1 | 2019 | Thailand | rectal swab | | clinical | Chromosome | 332 | One:Kne |
| GCF_019272405.1 | 2019 | Thailand | rectal swab | | clinical | Chromosome | 332 | One:Kne |
| GCF_019272495.1 | 2019 | Thailand | rectal swab | | clinical | Chromosome | 332 | One:Kne |
| GCF_019272575.1 | 2019 | Thailand | rectal swab | | clinical | Chromosome | 332 | One:Kne |
| GCF_019272675.1 | 2019 | Thailand | rectal swab | | clinical | Chromosome | 332 | One:Kne |
| GCF_019272715.1 | 2019 | Thailand | rectal swab | | clinical | Chromosome | 332 | One:Kne |
| GCF_019272755.1 | 2019 | Thailand | rectal swab | | clinical | Chromosome | 332 | One:Kne |

| NCBI RefSeq ID | year | nation | source | host | type | AssemblyLevel | mlst | serotype |
|---|---|---|---|---|---|---|---|---|
| GCF_019272875.1 | 2019 | Thailand | swab | | clinical | Chromosome | 332 | One:Kne |
| GCF_019273695.1 | 2019 | Thailand | food | | env/food | Chromosome | - | One:Knt |
| GCF_019321785.1 | 2018 | China | | *Penaeus vannamei* | env/food | Complete Genome | 1 | O1:Knt |
| GCF_020041945.1 | 2021 | South Korea | *Penaeus vannamei* | | env/food | Complete Genome | 413 | O1:Knt |
| GCF_021729965.1 | 2017 | China | | *Penaeus vannamei* | env/food | Complete Genome | 424 | O3:pK31 |
| GCF_021729985.1 | 2017 | China | | *Penaeus vannamei* | env/food | Complete Genome | 1800 | O1:pK33 |
| GCF_021730005.1 | 2016 | China | | *Penaeus vannamei* | env/food | Complete Genome | 1 | O1:pK1 |
| GCF_021730025.1 | 2017 | China | | *Penaeus vannamei* | env/food | Complete Genome | 424 | O3:pK31 |
| GCF_021730065.1 | 2015 | China | | *Penaeus vannamei* | env/food | Complete Genome | 1 | O1:pK1 |
| GCF_021730085.1 | 2015 | China | | *Penaeus vannamei* | env/food | Complete Genome | 122 | O2:Knt |
| GCF_023205895.1 | 2019 | China | seawater | | env/food | Complete Genome | 1805 | O3:Knt |
| GCF_023205915.1 | 2019 | China | seawater | | env/food | Complete Genome | 1799 | pO12:Knt |
| GCF_023205955.1 | 2018 | China | seawater | | env/food | Complete Genome | 722 | O11:Knt |
| GCF_023206515.1 | 2018 | China | seawater | | env/food | Complete Genome | 1750 | O11:K5 |
| GCF_025792495.1 | 2021 | China | feces | *Homo sapiens* | clinical | Chromosome | 3 | One:Kne |
| GCF_025917705.1 | 2016 | Germany | oyster | | env/food | Complete Genome | - | O5:K3 |
| GCF_025917745.1 | 2022 | China | | *Penaeus vannamei* | env/food | Complete Genome | - | O1:Knt |
| GCF_025920025.1 | 2019 | Germany | oyster | | env/food | Complete Genome | 1159 | O8:Knt |
| GCF_025920045.1 | 2017 | Germany | oysters | | env/food | Complete Genome | 1159 | O8:Knt |
| GCF_026650865.1 | 2020 | China | shrimp pond | | env/food | Complete Genome | - | O11:Knt |
| GCF_026970915.1 | 2020 | China | shrimp farm | *Penaeus vannamei* | env/food | Complete Genome | - | O1:K36 |
| GCF_028228685.1 | 2021 | Thailand | stool | *Homo sapiens* | clinical | Complete Genome | 3 | Ont:pK4 |
| GCF_028472865.1 | 2015 | China | shellfish | | env/food | Complete Genome | 864 | O8:K41 |
| GCF_028551295.1 | 2022 | China | | shrimp | env/food | Complete Genome | 1166 | O1:Knt |
| GCF_028609665.1 | 2016 | China | shrimp | | env/food | Complete Genome | - | O6:Knt |
| GCF_028609685.1 | 2014 | China | *Litopenaeus vannamei* | | env/food | Complete Genome | 424 | O3:pK31 |
| GCF_028609705.1 | 2014 | China | sediment | | env/food | Complete Genome | 424 | O3:pK31 |
| GCF_030060495.1 | 2021 | China | not applicable | fecal | clinical | Chromosome | 3 | One:Kne |
| GCF_030064615.2 | 2015 | Philippines | shrimp pond | *Penaeus vannamei* | env/food | Complete Genome | 1166 | O1:Knt |
| GCF_030064635.1 | 2015 | Philippines | shrimp pond | *Penaeus vannamei* | env/food | Complete Genome | 230 | pO12:Knt |

| NCBI RefSeq ID | year | nation | source | host | type | AssemblyLevel | mlst | serotype |
|---|---|---|---|---|---|---|---|---|
| GCF_030238725.2 | 2019 | Vietnam | | *Penaeus vannamei* | env/food | Complete Genome | 234 | O1:Knt |
| GCF_030291875.1 | 2015 | China | gastroenteritis | | clinical | Complete Genome | 224 | O4:K11 |
| GCF_030552955.1 | 2009 | China | | *Homo sapiens* | clinical | Complete Genome | 3 | O3:K6 |
| GCF_030704285.1 | 2022 | Malaysia | | prawn | env/food | Complete Genome | - | O10:Knt |
| GCF_030994145.1 | 2021 | Colombia | | *Anadara tuberculosa* | env/food | Complete Genome | - | O5:Knt |
| GCF_030994165.1 | 2021 | Colombia | | *Anadara tuberculosa* | env/food | Complete Genome | - | O1:Knt |
| GCF_030994185.1 | 2021 | Colombia | | *Anadara tuberculosa* | env/food | Complete Genome | - | O5:Knt |
| GCF_030994225.1 | 2021 | Colombia | | *Anadara tuberculosa* | env/food | Complete Genome | - | O5:K17 |
| GCF_030994565.1 | 2021 | Colombia | | *Anadara tuberculosa* | env/food | Complete Genome | - | O4:Knt |
| GCF_030998145.1 | 2021 | Colombia | | *Anadara tuberculosa* | env/food | Complete Genome | - | Ont:pK32 |
| GCF_033100075.1 | 2023 | China | water | | env/food | Complete Genome | 1160 | Ont:pK32 |
| GCF_033441435.1 | 2013 | Mexico | shrimp hepatopancreas | *Penaeus vannamei* | env/food | Complete Genome | 1525 | O6:Knt |
| GCF_033441455.1 | 2013 | Thailand | shrimp | *Penaeus vannamei* | env/food | Complete Genome | 970 | O1:Knt |
| GCF_033573735.1 | 2018 | China | | shrimp | env/food | Complete Genome | 452 | O6:Knt |
| GCF_033675235.1 | 2010 | China | shrimp | *Penaeus vannamei* | env/food | Chromosome | 114 | O5:Knt |
| GCF_034480095.1 | 2021 | Colombia | *Anadara tuberculosa* | | env/food | Complete Genome | - | O5:Knt |
| GCF_034480135.1 | 2021 | Colombia | *Anadara tuberculosa* | | env/food | Complete Genome | - | O1:Knt |
| GCF_034480485.1 | 2021 | Colombia | *Anadara tuberculosa* | | env/food | Complete Genome | - | Ont:pK32 |
| GCF_034480505.1 | 2021 | Colombia | *Anadara tuberculosa* | | env/food | Complete Genome | - | O5:K17 |
| GCF_036670155.1 | 2021 | Colombia | | *Anadara tuberculosa* | env/food | Complete Genome | - | O4:Knt |
| GCF_038050815.1 | 2020 | China | | *Penaeus vannamei* | env/food | Complete Genome | - | O1:K36 |
| GCF_038050985.1 | 2020 | China | | *Penaeus vannamei* | env/food | Complete Genome | - | O1:K36 |
| GCF_038070605.1 | 2022 | China | shrimp farm | *Penaeus vannamei* | env/food | Complete Genome | 415 | O3:Knt |
| GCF_038253855.1 | 2021 | Colombia | | *Anadara tuberculosa* | env/food | Complete Genome | - | O5:Knt |
| GCF_038741195.1 | 2019 | China | shrimp farm | *Penaeus vannamei* | env/food | Complete Genome | - | O1:K36 |
| GCF_039658515.1 | 2021 | China | seafood | | env/food | Complete Genome | - | O2:K28 |
| GCF_039658525.1 | 2018 | China | shrimp farm | *Penaeus vannamei* | env/food | Complete Genome | 1166 | O1:Knt |
| GCF_039658545.1 | 2019 | China | shrimp farm | *Penaeus vannamei* | env/food | Complete Genome | 180 | O1:Knt |
| GCF_040126105.1 | 2018 | China | shrimp farm | *Penaeus vannamei* | env/food | Complete Genome | 1166 | O1:Knt |
| GCF_040254425.1 | 1993 | Taiwan | | *Homo sapiens* | clinical | Complete Genome | - | O2:K28 |

| NCBI RefSeq ID | year | nation | source | host | type | AssemblyLevel | mlst | serotype |
|---|---|---|---|---|---|---|---|---|
| GCF_946810915.1 | 2002 | France | environmental | *Mytilus edulis* | env/food | Chromosome | - | O3:Knt |
| GCF_946810965.1 | 2005 | France | environmental | Seawater | env/food | Chromosome | 1294 | O11:Knt |
| GCF_946811045.1 | 2010 | France | environmental | Seawater | env/food | Chromosome | - | O4:Knt |
| GCF_946812515.1 | 1999 | France | environmental | *Mytilus edulis* | env/food | Chromosome | - | O1:Knt |
| GCF_946813825.1 | 2004 | France | clinical | *Homo sapiens* | clinical | Chromosome | 3 | O3:K6 |
| GCF_946814395.1 | 2009 | France | environmental | *Mytilus edulis* | env/food | Chromosome | - | O5:K17 |
| GCF_946814515.1 | 2009 | France | environmental | *Mytilus edulis* | env/food | Chromosome | - | O5:K17 |
| GCF_946814595.1 | 2014 | France | environmental | | env/food | Chromosome | 1140 | O4:Knt |

ENV, environment; AHPND, acute hepatopancreatic necrosis disease

**Table S2.** Results of Serotype Analysis Using an Alternative Serotype-related loci Sequence-Based Method for *Klebsiella pneumoniae* and *Acinetobacter baumannii*

| Sample | Best match locus | Best match type | Match confidence | Identity | Coverage | Length discrepancy | Expected genes in locus |
|---|---|---|---|---|---|---|---|
| Reference | KL6 | unknown (KL6) | Typeable | 99.99% | 100.00% | 8 bp | 13 / 23 (56.52%) |
| VP1909067_Contigs_Shovill | KL9 | unknown (KL9) | Typeable | 99.92% | 100.00% | 4 bp | 18 / 29 (62.07%) |
| VP2008484_Contigs_Shovill | KL156 | unknown (KL156) | Untypeable | 97.91% | 60.20% | -1319 bp | 10 / 20 (50.00%) |
| VP2009082_Contigs_Shovill | KL45 | unknown (KL45) | Typeable | 99.66% | 100.00% | 0 bp | 14 / 22 (63.64%) |
| VP2009236_Contigs_Shovill | KL132 | unknown (KL132) | Typeable | 98.73% | 100.01% | n/a | 16 / 24 (66.67%) |
| VP2010016_Contigs_Shovill | KL66 | unknown (KL66) | Untypeable | 96.74% | 55.43% | 1549 bp | 6 / 15 (40.00%) |
| VP2011073_Contigs_Shovill | KL139 | unknown (KL139) | Typeable | 97.05% | 99.83% | 2502 bp | 9 / 15 (60.00%) |
| VP2011082_Contigs_Shovill | KL156 | unknown (KL156) | Typeable | 99.46% | 56.79% | n/a | 9 / 20 (45.00%) |
| VP2011087_Contigs_Shovill | KL12 | unknown (KL12) | Typeable | 99.78% | 99.99% | -12 bp | 18 / 32 (56.25%) |
| VP2100001_Contigs_Shovill | KL47 | unknown (KL47) | Typeable | 98.20% | 99.88% | -9 bp | 10 / 16 (62.50%) |
| VP2100002_Contigs_Shovill | KL47 | unknown (KL47) | Typeable | 98.15% | 99.80% | -25 bp | 10 / 16 (62.50%) |
| VP2109117_Contigs_Shovill | KL146 | unknown (KL146) | Typeable | 99.60% | 100.02% | -993 bp | 20 / 28 (71.43%) |
| VP2109148_Contigs_Shovill | KL150 | unknown (KL150) | Typeable | 98.94% | 99.95% | -240 bp | 18 / 28 (64.29%) |
| VP2109155_Contigs_Shovill | KL150 | unknown (KL150) | Typeable | 98.94% | 99.95% | -240 bp | 18 / 28 (64.29%) |
| VP2109166_Contigs_Shovill | KL146 | unknown (KL146) | Typeable | 99.54% | 100.00% | -972 bp | 20 / 28 (71.43%) |
| VP2109175_Contigs_Shovill | KL146 | unknown (KL146) | Typeable | 98.96% | 99.90% | -9 bp | 20 / 28 (71.43%) |
| VP2109205_Contigs_Shovill | KL123 | unknown (KL123) | Untypeable | 87.25% | 3.63% | -31261 bp | 1 / 27 (3.70%) |
| VP2109285_Contigs_Shovill | KL129 | unknown (KL129) | Typeable | 99.78% | 100.02% | 7 bp | 16 / 23 (69.57%) |
| VP2109306_Contigs_Shovill | KL67 | unknown (KL67) | Typeable | 95.77% | 71.39% | -320 bp | 13 / 25 (52.00%) |
| VP2109324_Contigs_Shovill | KL28 | unknown (KL28) | Typeable | 99.49% | 100.00% | 0 bp | 28 / 52 (53.85%) |
| VP2109341_Contigs_Shovill | KL6 | unknown (KL6) | Typeable | 99.13% | 100.08% | -1471 bp | 13 / 23 (56.52%) |
| VP2109348_Contigs_Shovill | KL156 | unknown (KL156) | Typeable | 98.98% | 56.95% | n/a | 9 / 20 (45.00%) |
| VP2110020_Contigs_Shovill | KL114 | unknown (KL114) | Untypeable | 92.00% | 7.44% | n/a | 3 / 29 (10.34%) |
| VP2207085_Contigs_Shovill | KL20 | unknown (KL20) | Typeable | 97.98% | 100.14% | 45 bp | 10 / 18 (55.56%) |
| VP2208160_Contigs_Shovill | KL15 | unknown (KL15) | Typeable | 99.81% | 99.90% | -6 bp | 11 / 16 (68.75%) |
| VP2208160-1_Contigs_Shovill | KL15 | unknown (KL15) | Typeable | 99.81% | 99.90% | -6 bp | 11 / 16 (68.75%) |
| VP2208401_Contigs_Shovill | KL149 | unknown (KL149) | Typeable | 99.70% | 100.00% | -1 bp | 10 / 18 (55.56%) |
| VP2307007_Contigs_Shovill | KL32 | unknown (KL32) | Typeable | 97.32% | 100.00% | -298 bp | 13 / 19 (68.42%) |

| | | | | | | | |
|---|---|---|---|---|---|---|---|
| VP2307008_Contigs_Shovill | KL32 | unknown (KL32) | Typeable | 97.32% | 100.00% | -298 bp | 13 / 19 (68.42%) |
| VP2307019_Contigs_Shovill | KL156 | unknown (KL156) | Untypeable | 94.73% | 50.37% | 15644 bp | 7 / 20 (35.00%) |
| VP2307023_Contigs_Shovill | KL17 | unknown (KL17) | Typeable | 99.70% | 99.99% | 2 bp | 17 / 25 (68.00%) |
| VP2307090_Contigs_Shovill | KL17 | unknown (KL17) | Typeable | 100.00% | 100.00% | 0 bp | 17 / 25 (68.00%) |
| VP2307331_Contigs_Shovill | KL61 | unknown (KL61) | Typeable | 99.33% | 100.00% | -1 bp | 8 / 15 (53.33%) |
| VP2308037_Contigs_Shovill | KL17 | unknown (KL17) | Typeable | 99.70% | 99.99% | 2 bp | 17 / 25 (68.00%) |
| VP2308314_Contigs_Shovill | KL46 | unknown (KL46) | Typeable | 99.35% | 100.00% | 1033 bp | 12 / 21 (57.14%) |
| VP2309198_Contigs_Shovill | KL69 | unknown (KL69) | Typeable | 98.52% | 99.87% | -62 bp | 16 / 24 (66.67%) |
